# Beyond opioids: revisiting pain management in post cardiac surgery infants

**DOI:** 10.1186/s13054-024-04983-3

**Published:** 2024-06-07

**Authors:** Siddannagoud Salotagi, Atul Jindal

**Affiliations:** Division of Pediatric Pulmonology and Critical Care, Department of Pediatrics, All India Institute of Medical Sciences, Raipur, India

**Keywords:** Morphine, Paracetamol, Analgesia, Sedation, Pain scales

Dear Editor,

The article titled “Intermittent intravenous paracetamol versus continuous morphine in infants undergoing cardiothoracic surgery: a multi-center randomized controlled trial” by G Zeilmaker-Roest et al. was read with great interest [1]. We appreciate the authors’ research and want to express our views about the article.

Perioperative pain control is an essential component in post-operative intensive care. Inadequately treated pain may have both short-term and long-term deleterious effects. An evidence-based guideline for pain treatment after cardiac surgery in children is lacking. Acetaminophen and nonsteroidal anti-inflammatory drugs (NSAIDs) represent the most used non-opioid analgesics in children. In critically ill and post-operative children with moderate to severe pain, the SCCM PANDEM 2022 guideline recommends opioids as the primary analgesic [2]. Acetaminophen and nonsteroidal anti-inflammatory drugs (NSAIDs) are also suggested as an adjunct to reduce opioid usage and enhance early postoperative recovery [2].

Opioids have been associated with potentially serious adverse events, such as hypotension and respiratory depression, apart from the risk of tolerance and withdrawal. This study aims to look for whether paracetamol can be used as an alternative. There was a significant difference in the cumulative dose of morphine in both groups with nearly 5 times less in the paracetamol group. However, the study could not find any differences in hemodynamic instability, apnoeic episodes, delirium, or pain control. A mere decrease in the cumulative dose of morphine may not be a clinically meaningful outcome if there are no differences in major clinical outcomes. Hence authors could have used a better clinically significant primary outcome.

The study used NRS -11 (Numeric rating scale) to assess pain control and the need for a bolus dose of morphine in case of inadequate pain control. Though the Comfort B score was also assessed, it was not used to identify the need for intermittent morphine boluses. NRS -11 is not a validated score below 6 years of age especially in a preverbal stage as they will not be able to express [3]. The study states that trained pediatric ICU nurses and parents assessed the score; it is not clear what objective parameters were taken into consideration for scoring and the participation of parents without much training in such a short duration of stay to decide an intervention is also questionable.

According to this study, 62% and 69% of the paracetamol and morphine groups, respectively, required rescue morphine doses. Patients in the morphine group needed higher rescue doses of morphine raising concern for the use of opioids as the primary analgesic in sick children [1]. Despite having a lesser risk for sedation, morphine is still known to cause sedation at higher blood levels especially when used in conjunction with other sedatives [4]. The cumulative dose of morphine in the morphine group was much higher than that of the paracetamol group. It was a bit surprising that there was no difference in sedation requirement in both groups. The study also assessed delirium and gastrointestinal side effects in the first 48 h, as the author mentions the effects might be masked due to shorter follow-up. Considering these are very common adverse effects even with a short course of opioids this study could have followed up these children for a longer time to look for delirium and withdrawal syndromes [5].

This study also found higher renal injury in the morphine group, though it was statistically not significant. Opioids are known to cause podocytopathy in chronic usage and result in AKI if acute overdosage. The mechanism of AKI in such patients is due to dehydration, hypotension, rhabdomyolysis, and urinary retention^6^. There were no such differences in such adverse events in both groups. The study mentions such difference is probably due to the renal protective effect of paracetamol which is not proven in children and needs more studies for same.

We appreciate the authors for their study of using paracetamol as an alternative to opioids in post-cardiac surgery infants. The study also emphasized the role of cardiopulmonary bypass, hypothermia, and post-cardiac surgery state on the pharmacokinetics and pharmacodynamics of opioids. Currently, opioids being used as the first choice as an analgesic in critically sick infants, NSAIDS, Paracetamol or any other analgesic with a different mechanism should be used as an adjunct or even alternative to opioids to reduce the side effects associated. We need more research to find a better choice of analgesic in post cardiac surgery patients which are not affected by CPB with better safety profile.

## Data Availability

Not applicable.

## Abbreviations

AKI: Acute kidney injury
CPB: Cardiopulmonary bypass
NSAIDs: Non-steroidal anti-inflammatory drugs
NRS: Numeric rating scale
SCCM: Society of Critical Care Medicine

## References

[1] Zeilmaker-Roest G, de Vries-Rink C, van Rosmalen J, van Dijk M, de Wildt SN, Knibbe CA (2024). Intermittent intravenous paracetamol versus continuous morphine in infants undergoing cardiothoracic surgery: a multi-center randomized controlled trial. Crit Care.

[2] Smith HA, Besunder JB, Betters KA, Johnson PN, Srinivasan V, Stormorken A (2022). 2022 Society of Critical Care Medicine clinical practice guidelines on prevention and management of pain, agitation, neuromuscular blockade, and delirium in critically ill pediatric patients with consideration of the ICU environment and early mobility. Pediatr Crit Care Med.

[3] Giordano V, Edobor J, Deindl P, Wildner B, Goeral K, Steinbauer P (2019). Pain and sedation scales for neonatal and pediatric patients in a preverbal stage of development: a systematic review. JAMA Pediatr.

[4] Morphine sulfate injection label—accessdata.fda.gov Reference ID: 3043802

[5] Ávila-Alzate JA, Gómez-Salgado J, Romero-Martín M, Martínez-Isasi S, Navarro-Abal Y, Fernández-García D (2020). Assessment and treatment of the withdrawal syndrome in paediatric intensive care units: systematic review. Medicine.

[6] Mallappallil M, Bajracharya S, Salifu M, Yap E. Opioids and acute kidney injury. In Seminars in nephrology 2021 Jan 1 (Vol. 41, No. 1, pp. 11–18). WB Saunders. 10.1016/j.semnephrol.2021.02.002 PMC9579435 33896468

